# In Situ X-ray Absorption Spectroscopy Study of the Deactivation Mechanism of a Ni-SrTiO_3_ Photocatalyst Slurry Active in Water Splitting

**DOI:** 10.1021/acs.jpcc.4c04688

**Published:** 2024-09-17

**Authors:** MemetTursun Abudukade, Marco Pinna, Davide Spanu, Giuditta De Amicis, Alessandro Minguzzi, Alberto Vertova, Sandro Recchia, Paolo Ghigna, Guido Mul, Marco Altomare

**Affiliations:** † Department of Chemical Engineering, MESA+ Institute for Nanotechnology, University of Twente, P.O. Box 217, Enschede 7500 AE, The Netherlands; ‡ Department of Science and High Technology, University of Insubria, Via Valleggio 11, Como 22100, Italy; § Dipartimento di Chimica, Università degli Studi di Milano, Via Golgi 19, Milan 20133, Italy; ∥ Dipartimento di Chimica, Università degli Studi di Pavia, Via Taramelli 16, Pavia 27100, Italy; ⊥ UdR INSTM di Milano - Consorzio Interuniversitario Nazionale per la Scienza e Tecnologia dei Materiali − INSTM, Via G. Giusti 9, Firenze 50121, Italy

## Abstract

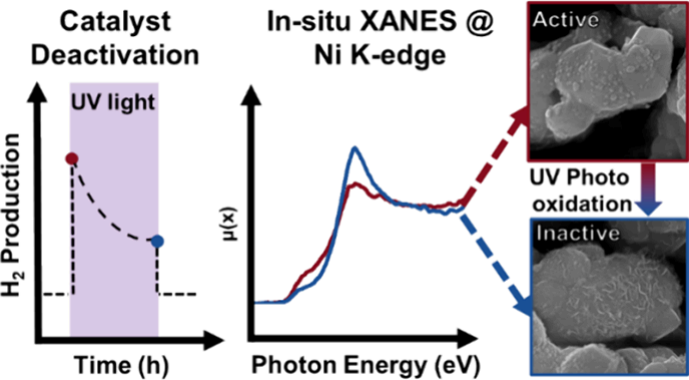

We used in situ X-ray absorption spectroscopy (XAS) to investigate the composition–performance correlation of Ni-SrTiO_3_ photocatalysts active for water splitting. After preparation and exposure to ambient conditions, the Ni particles on SrTiO_3_ consist of Ni(0) and Ni(II) phases, with a 4:1 at % ratio, in a metal/oxide core/shell configuration, as confirmed by XPS and TEM-EDX. In situ XAS experiments using an aqueous slurry of the Ni-SrTiO_3_ photocatalyst and simultaneous continuous exposure to 365 nm light with a power density of 100 mW cm^–2^ and the X-rays do not reveal significant changes in oxidation state of the Ni particles. Contrarily, when the X-rays are discontinuously applied, UV excitation leads to oxidation of a significant fraction of Ni(0) to Ni(II), specifically to NiO and Ni(OH)_2_ phases, along with cocatalyst restructuring. Ni dissolution or oxidation to higher valence states (e.g., Ni(III)) was not observed. The UV light-induced oxidation of Ni(0) causes the hydrogen evolution rate to drop to similar rates as observed for pristine SrTiO_3_, suggesting that Ni(0) is the active phase for H_2_ generation. Our results underscore the importance of assessing the effects of (continuous) X-ray exposure to (photo)catalyst-containing aqueous slurries during in situ XAS experiments, which can significantly influence the observation of compositional and structural changes in the (photo)catalysts. We ascribe this to X-ray induced water photolysis and formation of free electrons, which in this study quench SrTiO_3_ photoholes and prevent Ni oxidation.

## Introduction

In heterogeneous photocatalysis, semiconductors such as metal oxides are commonly used to absorb UV–visible light and generate charge carriers, i.e., conduction band (CB) electrons and valence band (VB) holes. Once separated, photogenerated charge carriers diffuse toward the surface of the photocatalyst, where they participate in redox reactions.^1−4^ Strontium titanate (SrTiO_3_, herein STO) is one of the most studied photocatalysts in photocatalytic water splitting due to its stability under illumination in aqueous electrolytes and suitable band edge positions that allow for both water reduction to H_2_ and water oxidation to O_2_.^5^ However, bare STO exhibits poor photocatalytic activity, likely due to sluggish kinetics of charge carrier transfer at the semiconductor/electrolyte interface. To overcome this issue, cocatalysts are typically deposited on the surface of semiconductors.^6,7^ Noble metals are considered benchmark cocatalysts for proton or water reduction (e.g., Pt or Rh) and their oxides for oxidation of water (e.g., Ru or Ir oxides).^8−10^ However, their scarcity and high cost have directed research toward alternative earth-abundant metals.^11−13^ Among these, Ni-based materials are an attractive option.^14−17^ In alkaline electrolysis, Ni is an efficient catalyst for the hydrogen evolution reaction (HER),^18,19^ while NiOOH is active for the oxygen evolution reaction (OER).^20,21^

STO photocatalysts, surface-modified with various Ni-based cocatalysts, have been widely investigated in the last decades.^15,17,22−26^ Earlier studies applying a relatively long time resolution (hours) for analysis of H_2_ and O_2_ evolution^25^ reveal little changes in performance over time, while recent studies applying gas chromatography with minute-time resolution and ppm-level detectors reveal significant changes in H_2_ evolution rate and H_2_:O_2_ ratio in the first hour(s) of exposure to UV illumination.^15,16^

The function of the NiO shell in NiO/Ni particles, present after preparation of the catalyst, was proposed by Domen et al. to promote the HER, with the metallic Ni core providing ohmic contact and hence electron transfer from STO to the NiO shell.^22^ It was also proposed by the same authors that water oxidation is catalyzed by the free STO surface.^22^ On the contrary, Baba and Fujishima proposed that the cathodic site for hydrogen evolution is the bare STO surface.^27^ Finally, Townsend and colleagues suggested that in NiO/Ni-STO photocatalysts, Ni is the HER catalyst, and NiO the OER site.^17^ In most of these studies, the Ni-STO photocatalysts were photoexcited using simulated solar light and, hence, with relatively low UV photon fluxes.

Structural changes in the NiO/Ni particles have also been observed on the shorter time scales of light exposure (<1 h) and have been correlated with changes in performance. Zhang et al. reported on deactivation of the photocatalyst and assigned this to a photoinduced oxidation/dissolution of the metallic core in the NiO/Ni cocatalyst, similarly to what was posited by Townsend et al.^16^ Han et al. investigated the transient behavior of NiO/Ni-STO under intermittent illumination. They proposed a mechanism based on three redox steps: (i) the light-driven oxidation of surface Ni(OH)_2_ to trivalent Ni species (NiOOH) and protons (H^+^), as reported in eq 1, (ii) proton reduction on metallic Ni sites (eq 2), and (iii) the reaction of NiOOH and Ni to Ni(OH)_2_ (eq 3) under dark conditions, leading to catalyst regeneration.^15^ A more recent study from the same authors shows that deposition of CrO_*x*_ improves the stability of the NiO/Ni cocatalyst on Mg-doped STO due to formation of a mixed metal oxide phase (NiMgCrO_*x*_), which is proposed to be the active site for HER.^28^
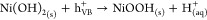
1
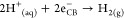
2
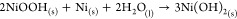
3

All these propositions for composition and structural changes, however, were based on ex-situ characterization techniques and should be analyzed critically as photocatalysts were exposed to environmental conditions after photocatalysis. Thus, the Ni speciation measured by ex-situ methods (e.g., XPS, XAS) after photocatalysis might not be fully representative of the photocatalyst state during photocatalysis.

Some of us previously used operando X-ray absorption spectroscopy (XAS) techniques to investigate the dynamic behavior of Ni (and Cu) cocatalyst nanoparticles (NP) on TiO_2_ in photocatalytic H_2_ generation from alcoholic aqueous mixtures.^29^ We observed cocatalyst dissolution in the dark (forming Ni^2+^ (aq.) or Cu^2+^ (aq.) species), followed by a UV-light-induced healing of the cocatalyst, i.e., photodeposition onto TiO_2_ of metallic Ni or Cu from solvated Ni^2+^ or Cu^2+^ species, causing a gradual increase of the H_2_ evolution rate. In the presence of a hole scavenger (i.e., when holes are rapidly consumed), metallic Ni (or Cu) is the active site for H_2_ evolution. In a more recent study, simple illumination of slurries of reduced TiO_2_ powders in the presence of a dissolved Ni(II) salt was found to form metastable Ni(I) species active toward H_2_ generation, as probed by in situ XAS and EPR spectroscopy techniques.^30^

In the present work, we study the composition–performance correlation of a Ni-STO photocatalyst for overall water splitting under UV exposure (365 nm, 100 mW cm^–2^), in the absence of sacrificial species, focusing on in situ X-ray spectroscopy. For the photocatalytic water splitting tests, we used a continuously stirred tank photoreactor connected to an online gas chromatograph (micro-GC, with PDD), providing a high time resolution of 2 min. We critically assess in situ XAS experiments and demonstrate that X-ray exposure should be minimized to observe UV light-induced composition and structural changes of the Ni-STO photocatalyst. In particular, we reveal oxidation of the metallic Ni phase, within a few hours of UV illumination, to Ni(II) species, namely, NiO and Ni(OH)_2_ solid phases. Given the concurrent decrease in H_2_ evolution rate, we infer that the metallic nickel (Ni(0)) is the cocatalyst phase active for H_2_ generation.

## Methods

### Preparation of Ni-STO

The Ni-STO photocatalyst was prepared by following a recipe reported in a previous study.^15^ STO was synthesized by a high-temperature solid-state synthesis. Stoichiometric amounts of SrCO_3_ (99.995%, Sigma-Aldrich) and rutile TiO_2_ (99.995%, Sigma-Aldrich) were mixed by grinding in an agate mortar for 15 min, placed in a ceramic sample-holder, and calcined at 1000 °C (heating rate 10 °C min^–1^) for 1 h in static air. To obtain a nominal loading of 3 wt % of Ni cocatalyst NPs on STO, we employed a wet impregnation method using a Ni salt (NiNO_3_ · 6H_2_O) following a recipe previously reported.^31^ To do so, 200 mg of synthesized STO powders was dispersed in 20 mL of a 3.95 mM Ni(NO_3_)_2_ aqueous solution and stirred for 2 h. Then, the solution was evaporated in air at 80 °C overnight and transferred into a tube furnace for the following thermal treatment. In the tube furnace, the powders were first calcined at 400 °C in air (30 mL min^–1^) for 30 min, and then flushed in Ar (50 mL min^–1^) to cool down to room temperature. After reaching room temperature, the Ar flow was replaced by 5% H_2_ in Ar (30 mL min^–1^) and then the sample was heated to 500 °C for 5 h. The sample was then cooled to room temperature in a flow of Ar. Contrary to earlier studies, the sample was not exposed to a mild oxidation in air at 200 °C but carefully exposed to ambient conditions. Finally, the catalyst powder was ground and stored in a capped glass vial.

### Ex-Situ Characterization

The morphology of the Ni-STO photocatalyst powders was examined by field emission scanning electron microscopy (FE-SEM, JEOL, JSM-7610F) and high-angle annular dark-field imaging-transmission electron microscopy (HAADF-TEM, Thermo Scientific Spectra 300 S/TEM). The chemical composition of the cocatalyst was analyzed by energy-dispersive X-ray spectroscopy-transmission electron microscopy (EDX-TEM, Thermo Scientific Spectra 300 S/TEM) and X-ray photoelectron spectroscopy (XPS, PHI Quantes scanning XPS/HAXPES microprobe, Al Kα, 100 μm beam). CasaXPS software (Casa Software, Ltd.) was used for C 1s correction and fitting of the high-resolution Ni 2p spectra. Fitting of the experimental XPS spectra was performed by using Voigt curves and a Shirley background. X-ray diffraction was used to analyze the crystallographic properties of the photocatalyst powders (D2 PHASER, Bruker, Cu Kα source, step size 0.02°, 0.5 s/step, scan range 10–80°). Inductively coupled plasma mass spectrometry (ICP-MS, ICAP Q, Thermo Scientific) was used to evaluate the actual loading of Ni on STO, the molar ratio of Sr and Ti, and to investigate the possibility of Ni dissolution in water during the photocatalytic experiments. 9.97 mg of Ni-STO powders was added in a PTFE vessel along with 5 mL of trace-grade analysis hydrochloric acid (37% v/v, VWR). Microwave-assisted acid digestion was performed under temperature-controlled conditions at 190 °C for 6 h by employing an ETHOS One (Milestone) digestion system. After digestion, the samples were diluted and acidified with ultrapure nitric acid obtained by sub-boiling distillation.^32^ 30 g of digested solution was analyzed to determine the Ni loading and molar ratio of Sr and Ti. Photocatalyst suspensions (sampled after suspending the powders in water under dark or illumination conditions) were filtered and stabilized by acidification using nitric acid of trace-grade analysis quality (65%, Suprapur, Supelco). Diluted solutions were analyzed by ICP-MS.

### Photocatalytic Experiments

The photocatalytic activity of the Ni-STO powders was measured by using a continuously stirred tank reactor connected to an online gas chromatograph (GC) equipped with a pulsed discharge detector (GC-PDD, CompactGC 4.0, Interscience). See Figure S1 for a scheme and a photograph of the setup. The photoreactor was a gastight system based on an optical glass cuvette (402.013-OG, Hellma). The cuvette was filled with 25 mL of an aqueous catalyst suspension (slurry) with a concentration of photocatalyst of 1 mg mL^–1^ (e.g., 25 mg of Ni-STO in 25 mL of deionized (DI) water). This catalyst concentration is at the upper limit of the linearly increasing regime of the rate as a function of catalyst concentration.^33^ To disperse the photocatalysts, 25 mg of catalyst powder was added to 10 mL of DI water in a glass vial. To ensure complete retrieval of the photocatalyst, DI water was added to the glass vial and sonicated. This step was repeated twice up to a total volume of 25 mL. Once loaded in the reactor, the suspension was kept under vigorous stirring and illuminated with monochromatic UV light (LED Control 5S, Opsytec, λ = 365 nm, irradiance = 100 mW cm^–2^). A constant He flow (10 mL min^–1^) was bubbled through the suspension first to remove dissolved oxygen in the solution under dark conditions and then, under UV illumination, to purge the gaseous products (H_2_ and O_2_) to the GC for quantification. The time resolution of the online GC analysis is 2 min, and the response delay is 3 min. The production rate of H_2_ and O_2_ (μmol h^–1^ g_cat_^–1^) is calculated according to eq 4:
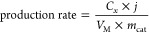
4where

- *C*_*x*_ is the concentration of evolved H_2_ or O_2_ gas expressed as part per million (μL L^–1^)
- *j* is the flow rate of carrier gas (He) expressed in L h^–1^
- *V*_m_ is the molar volume at 298.73 K (24 μL μmol^–1^)
- *m*_cat_ is the mass of the catalyst expressed in grams

### In Situ XAS Experiments and Spectroscopic Cell Design

The in situ XAS experiments were conducted at beamline P65 (PETRA III storage ring) at DESY, Hamburg, Germany, and at beamline XAFS at ELETTRA, Trieste, Italy. For the in situ XAS experiments, we used a liquid-phase cell as shown in Figure S2a, similar to that used in a previous work.^29^ This cell has a thin Mylar front window (01867-AB, SPI, 6 μm) to provide transparency for both X-ray and UV light and is gastight, allowing for online analysis of the gas head space by GC. The photocatalyst suspension, kept under constant stirring, was purged continuously with Ar during the experiments to simulate the photocatalytic conditions adopted with the photocatalytic reactor (Figure S1), including the UV photon flux irradiated on the photocatalyst slurry. We performed in situ experiments in fluorescence mode, while reference samples (metallic Ni, NiO powder, Ni(OH)_2_ powder, and aqueous NiSO_4_ solution) and pellets of as-prepared Ni-STO powder were analyzed in transmission mode. X-ray absorption near edge structure (XANES) spectra were collected in the −150 to +150 eV photon energy range with respect to the Ni K-edge (8333 eV), allowing to record one spectrum every ∼3 min and hence to carry out time-resolved experiments. To minimize the effect of beam damage (discussed in Results and Discussion), the exposure of the photocatalyst slurry to the probing X-ray beam was systematically reduced in a series of in situ experiments; while this approach reduced the time resolution, it allowed to follow Ni redox processes caused by UV excitation of Ni-STO. Quantitative analysis of the different Ni species in the cocatalyst was obtained by fitting the acquired spectra using linear combinations of reference spectra with the Athena software.^34^

## Results and Discussion

**Figure 1:**
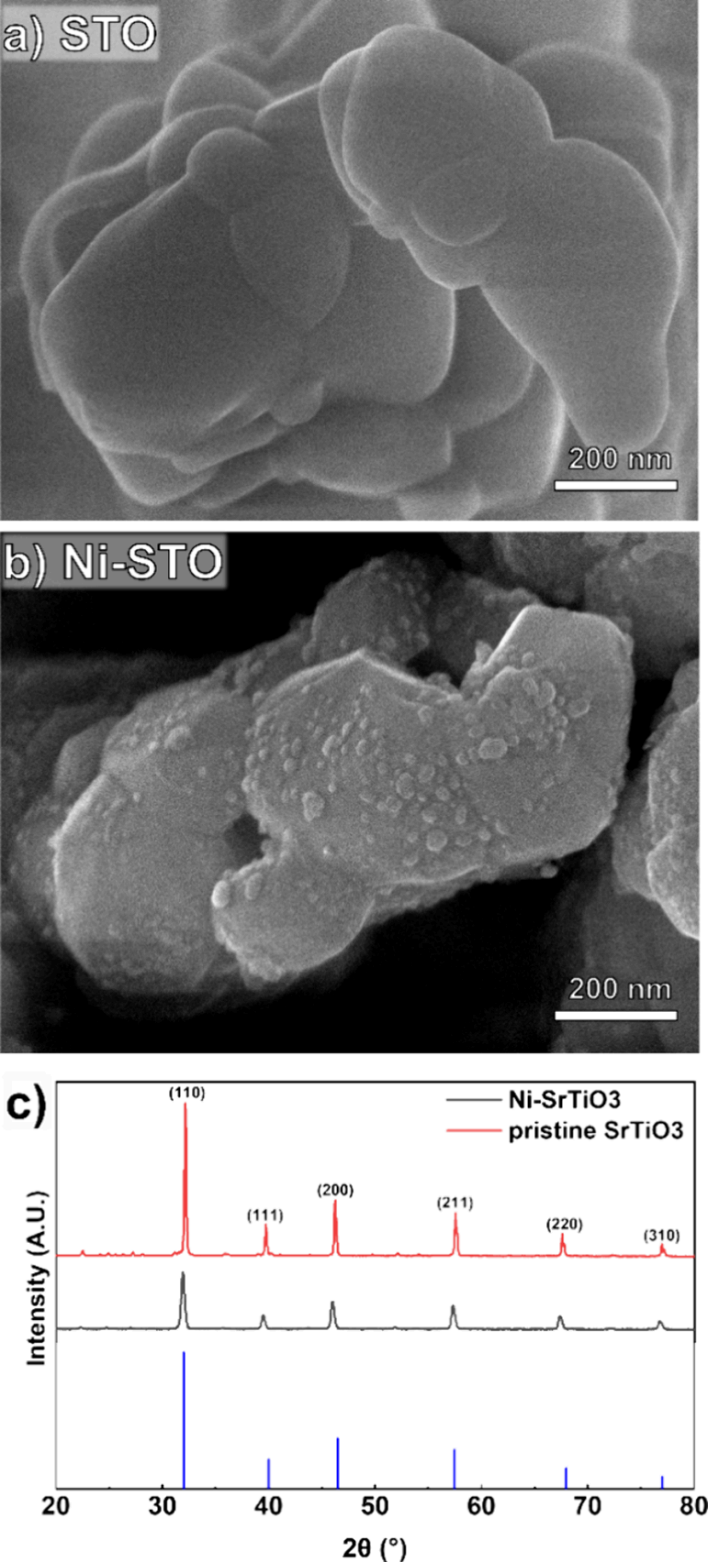
High-resolution SEM images of (a) pristine STO and (b) Ni-STO. (c) XRD patterns of pristine STO and Ni-STO.

The morphology of bare STO and Ni-STO particles was analyzed by SEM. The STO powders consist of round-shaped, agglomerated particles in the size range of 100–500 nm (Figure 1a). Upon surface modification of STO, nanoparticles (NPs) of Ni in the size range of 10–30 nm are formed (Figure 1b). The ICP-MS measurements show a stoichiometric molar ratio between Sr and Ti (Sr:Ti = 1.0) and a 2.0 wt % loading of Ni. The crystalline nature of both pristine STO and Ni-modified STO is confirmed by XRD analysis (see XRD patterns in Figure 1c).^35^

TEM analysis (Figure 3a, to be discussed later) clearly shows that the Ni particles consist of Ni/NiO core–shell particles—similar to a previous study.^15^

**Figure 2:**
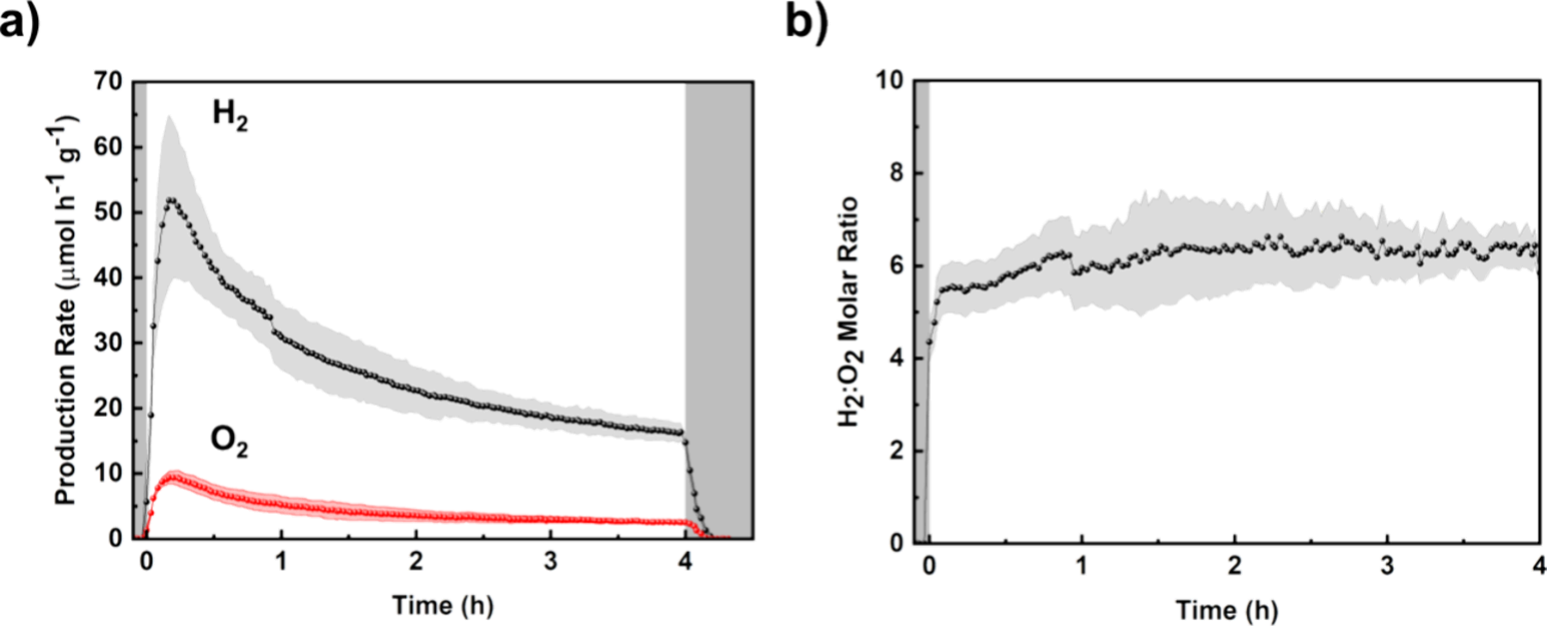
(a) Production rate profiles for H_2_ (black) and O_2_ (red) during overall photocatalytic water splitting. (b) Molar ratio of evolved H_2_ and O_2_. The light gray and red shaded areas represent the standard deviation calculated for the same experiment reproduced three times, and the gray and white areas represent times in the absence of UV illumination (dark condition) and UV irradiation steps, respectively.

The photocatalytic activity of Ni-STO was tested under intermittent, high-UV photon flux illumination (Figure 2). Soon after the UV illumination is switched on, both H_2_ and O_2_ production rates reached a maximum value of 50 ± 13 and 9 ± 1 μmol h^–1^ g^–1^, respectively (Figure 2a). This production rate is equivalent to an apparent quantum efficiency of ∼0.2%. This value is significantly lower than values reported in the literature, explained by the high intensity of UV light (100 mW cm^–2^) entering the reactor, equivalent to values in which the reaction rate likely no longer increases linearly as a function of photon flux. We applied high light intensities to accelerate possible composition and structural changes of the photocatalyst induced by UV light exposure. A continuous decrease in both H_2_ and O_2_ evolution rates can be observed, just after the peak in production rate is reached—this testifies the rapid photocatalyst degradation under high photon flux UV irradiation.

This, together with the observation that the molar ratio of H_2_:O_2_ is higher than 2:1 (Figure 2b), suggests that photogenerated holes in Ni-STO take part in other redox processes than just the OER, probably causing oxidation of the cocatalyst. Additional photocatalytic experiments using the same photocatalyst slurry were performed under intermittent UV illumination (light on/off cycles, Figure S3). The deactivation of the catalyst appears to occur in two phases: a relatively fast initial phase, followed by a slower secondary phase. As shown in Figure S3, when UV light is switched on again, after a dark period of 15 h, the hydrogen production rate peaks initially at the same value reached at the end of the first irradiation cycle and then decays in a similar manner. Hence, we can conclude that degradation of the photocatalyst takes place under UV illumination, while the resulting composition of Ni-STO is stable in water under dark conditions. This behavior is different from the study of Han and co-workers on a similar catalyst system,^15^ who observed significant recovery of the initial rate after applying a period in the dark—implying that our catalyst does not express the composition and structural changes observed by Han and co-workers.^15^

**Figure 3:**
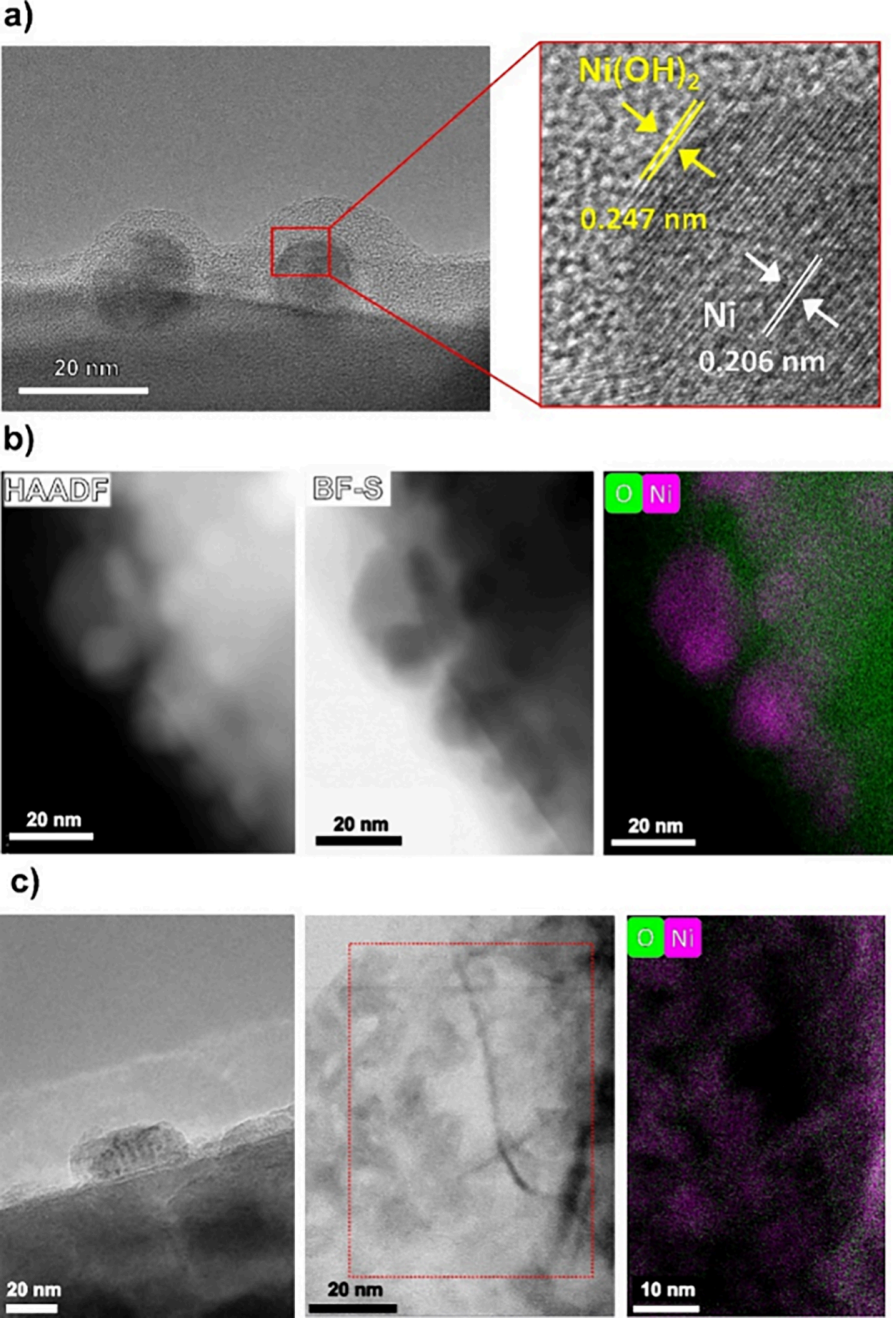
(a, b) As-prepared Ni-STO photocatalyst: (a, left) TEM and (a, right) HR-TEM images; (b, left) HAADF and (b, center) BF-S images; (b, right) TEM-EDX elemental mapping. (c) Ni-STO photocatalyst after 6 h of photocatalysis: (c, left) TEM and (c, center) HR-TEM images and (c, right) TEM-EDX elemental mapping.

To reveal composition changes, we initially performed ex-situ TEM analysis. TEM images of the as-prepared sample (Figure 3a) show the core–shell nature of the Ni cocatalyst NPs. The Ni NPs in Figure 3a (left) have a diameter of ca. 10–15 nm (in line with the average NP diameter observed by SEM, Figure 1). The average NP shell thickness is approximately 2 nm (Figure 3a, right). The metallic nature (Ni(0)) of the core was confirmed by analysis of the lattice spacing, i.e., 0.206 nm, attributed to the Ni (111) planes, while the lattice spacing of 0.247 nm observed for the shell can be attributed to the (220) planes of NiO (Ni(II)).^36^ Elemental mapping of the NP via TEM-EDS measurements (Figure 3b) shows that both Ni and O are present in regions corresponding to the outer layer (shell) of the cocatalyst NPs, providing further proof of a metal/oxide core–shell structure.

**Figure 4:**
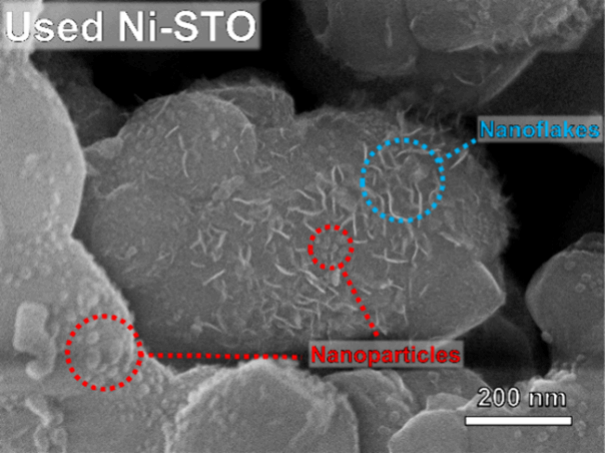
High-resolution SEM (HR-SEM) image of Ni-STO after 6 h of photocatalysis. For ease of visualization, some core–shell NPs have been highlighted in red, while flake-like nanostructures have been highlighted in blue.

After photocatalysis, Ni NPs with a rougher surface can be observed (Figure 3c), along with the presence of few nanometer-thick flake-like structures, also observable by SEM (below, Figure 4). The TEM-EDS elemental map of the flakes (Figure 3c) shows a homogeneous distribution of Ni and O, suggesting that such nanoflake formations are composed of an oxidized Ni phase, e.g., Ni(OH)_2_, which is known to grow in sheet/plate-like morphologies.^37^

**Figure 5:**
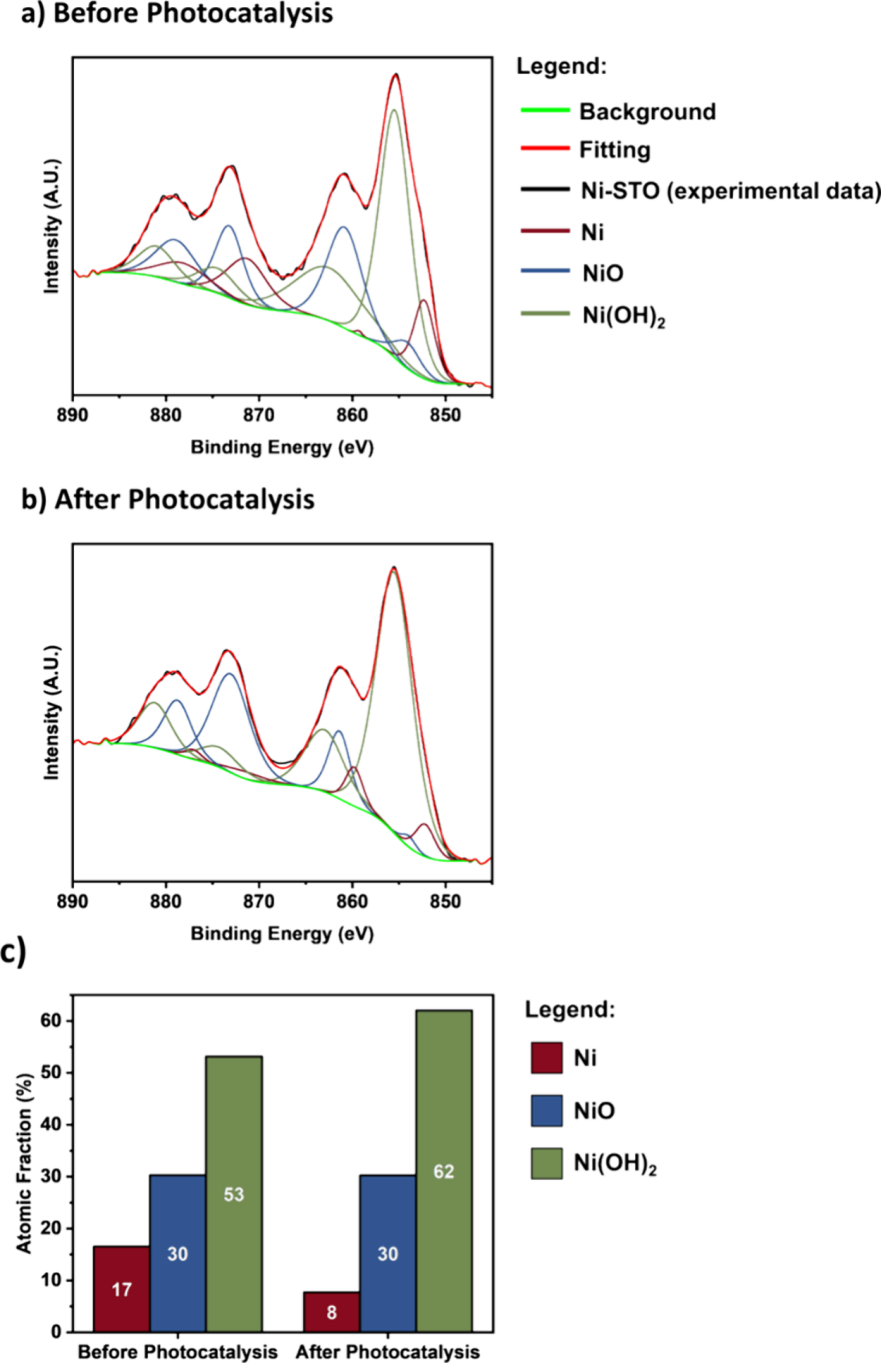
(a, b) Ni 2p HR-XPS spectra for the as-prepared sample and after 6 h of photocatalysis, respectively. (c) Relative surface composition of Ni before and after photocatalysis.

XPS measurements for the photocatalyst powders were performed before and after photocatalysis to assess possible changes in the oxidation state of the Ni cocatalyst (Figure 5). The Ni 2p spectra reveal the presence of metallic Ni (at 852.30 eV), NiO (at 854.10 eV), and Ni(OH)_2_ (at 855.42 eV) phases, attributed to electronic transitions from Ni 2p 1/2 and Ni 2p 3/2 levels, in agreement with previous literature.^38^ Quantitative analysis showed that Ni(OH)_2_ is present as the predominant species in both samples. This could be explained by the nature of XPS: being a surface-sensitive technique, with a probing depth in the nanometer range,^39^ only the outer layer of the NP can be probed by XPS (outermost ∼5 nm or so). Although a slight oxidization of metallic Ni after photocatalysis might have taken place (the fraction of Ni(OH)_2_ on the cocatalyst surface increased from 53 to 62%, Figure 5c), these minor changes cannot fully justify the significant deactivation observed with the photocatalytic measurements (Figure 2). Furthermore, XPS results indicate that cocatalyst dissolution did not take place during photocatalysis, as the surface atomic content of Ni (at%) for the samples before and after photocatalysis is identical within the experimental error of the applied technique (Table S1). These results are corroborated by ICP-MS data (Table S2), showing that suspending photocatalyst powders in DI water for up to 15 h in the dark, or under UV illumination for up to 6.5 h, only leads to dissolution of negligible amounts of nickel (typically in the order or below 0.1% of the Ni mass initially loaded on STO).

The composition of the Ni NP in the as-prepared photocatalyst was also measured by ex-situ XAS measurements. The fitting results show that the cocatalyst is mainly composed of metallic Ni (∼80%, NP core) with a ∼ 20% of Ni(II) phase, in the form of NiO and Ni(OH)_2_, accounting for the composition of the NP shell (see Figure S4 along with a brief description of the spectral shapes in the Supporting Information).

Most importantly, we performed in situ XAS experiments to investigate in real time changes in the Ni oxidation state during photocatalysis, under high-flux UV irradiation. To achieve temporal resolution in the range of minutes, spectra were acquired only in the XANES region. NiO and Ni(OH)_2_ phases cannot be easily discriminated by fitting XANES spectra. Thus, phase composition data are provided as the content of metallic Ni (Ni(0)) and Ni(II), where the content of Ni(II) includes both NiO and Ni(OH)_2_ phases. Analyzing the results using the reference spectrum of NiOOH as the fitting component resulted in a lower fitting quality of the in situ spectra (Figure S5). We therefore exclude the presence or formation of Ni(III) phases. Moreover, solvated Ni(II) species were also not identified, indicating no or negligible dissolution of the Ni cocatalyst, well in line with XPS and ICP-MS results.

During preliminary in situ XAS experiments (in DI H_2_O, under UV light on–off cycles), only negligible changes in Ni phase composition were observed both in the dark and under UV illumination conditions (Figure S6, discussed in the SI). Online GC analysis of the gas headspace from the in situ XAS cell in the absence of X-rays (Figure S7) provides H_2_ and O_2_ evolution profiles similar to those measured with the reactor applied in our lab (compare Figure S7 and Figure 2). That is, when UV is switched on, both H_2_ and O_2_ evolution profiles reach a maximum rate in a few minutes, followed by a rapidly decreasing H_2_ evolution rate. Hence, the in situ XAS data in Figure S6 cannot explain the photocatalyst deactivation; the significant decrease in hydrogen production rate and lack of changes in the composition of Ni/NiO core–shell particles are difficult to reconcile.

**Figure 6:**
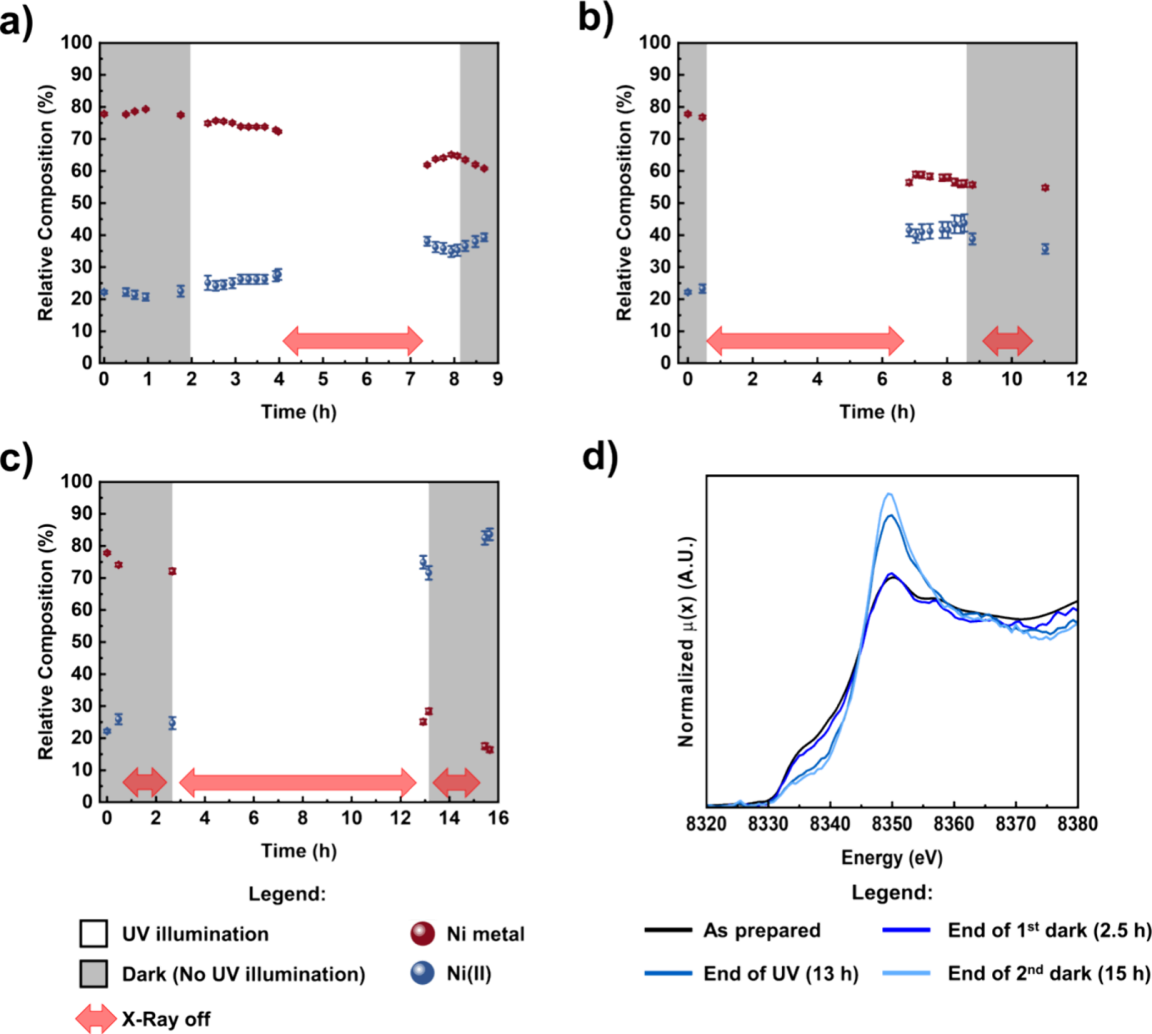
(a–c) Fitting results of in situ XANES spectra of Ni-STO with reduced exposure of the photocatalyst slurry to the probing X-ray beam. The data at *t* = 0 refer to the ex-situ XANES measurements of as-prepared Ni-STO; gray areas represent data points acquired under dark conditions, while white areas refer to data points acquired under UV illumination, and red arrows refer to the absence of X-ray (X-ray shutter closed). In (b), the exposure time to UV light amounts to ∼4 h, while in (c), the exposure time to UV light amounts to ∼10 h. (d) Selected, normalized Ni K-edge XANES spectra of Ni-STO for experiment (c).

Therefore, we performed a series of in situ experiments where the UV-illuminated photocatalyst slurry was exposed to the probing X-ray beam (for XAS spectra collection) for gradually shorter times (Figure 6).

In each experiment, prior to UV light illumination, no significant change of the Ni(0) and Ni(II) composition can be observed, the at % ratio remaining close to the initial one of 4:1. Moreover, photocatalytic experiments carried out after different durations of the initial He purging step in the dark show comparable H_2_ gas evolution profiles (Figure S8). This further confirms that the photocatalyst is stable in DI water in the dark, and no cocatalyst degradation takes place in the aqueous suspension before or in the absence of UV irradiation.

However, clear changes in the Ni oxidation state take place under UV illumination, as shown in Figure 6a–c, where each plot represents a single batch experiment. For example, the experiment in Figure 6a shows that after ca. 2 h of UV illumination with continuous X-ray exposure, minor but sizable changes in the Ni(0) and Ni(II) atomic fractions can be detected, well in line with data in Figure 5. However, when the photocatalyst is kept for another 3 h of UV illumination in the absence of X-ray exposure, the oxidation of Ni becomes even more noticeable, as the Ni(0):Ni(II) ratio decreases from 4 (80% Ni(0), 20% Ni(II)) to about 1.5 (60% Ni(0), 40% Ni(II))—shown in Figure 6b. Furthermore, Figure 6c demonstrates that extending the UV exposure time (in the absence of X-ray) leads to an inversion of the ratio of Ni(0):Ni(II) from 4 to 0.25, i.e., prolonged UV irradiation leads to further oxidation of the remainder Ni phase in the cocatalyst.

Figure 6d shows the XANES spectra after each light on–off cycle with minimized X-ray exposure of the suspension for the experiment in Figure 6c (10 h-long UV irradiation performed completely in the absence of X-ray exposure). The dramatic increase in the intensity of the white line in the Ni K-edge XANES spectra after UV illumination proves the formation of large amounts of oxidized nickel phases (solid NiO and Ni(OH)_2_). This correlates well with the results of ex-situ XPS, showing that the content of the Ni(OH)_2_ phase increases after photocatalysis. The quantitative difference in phase compositions from the two techniques (XAS vs XPS) can be explained by considering that XAS is a bulk technique, thus probing the entirety of the cocatalyst NPs. On the contrary, XPS is surface-sensitive (with an average penetration depth of a few nanometers), and hence, it allows to probe only the photocatalyst surface.

**Figure 7:**
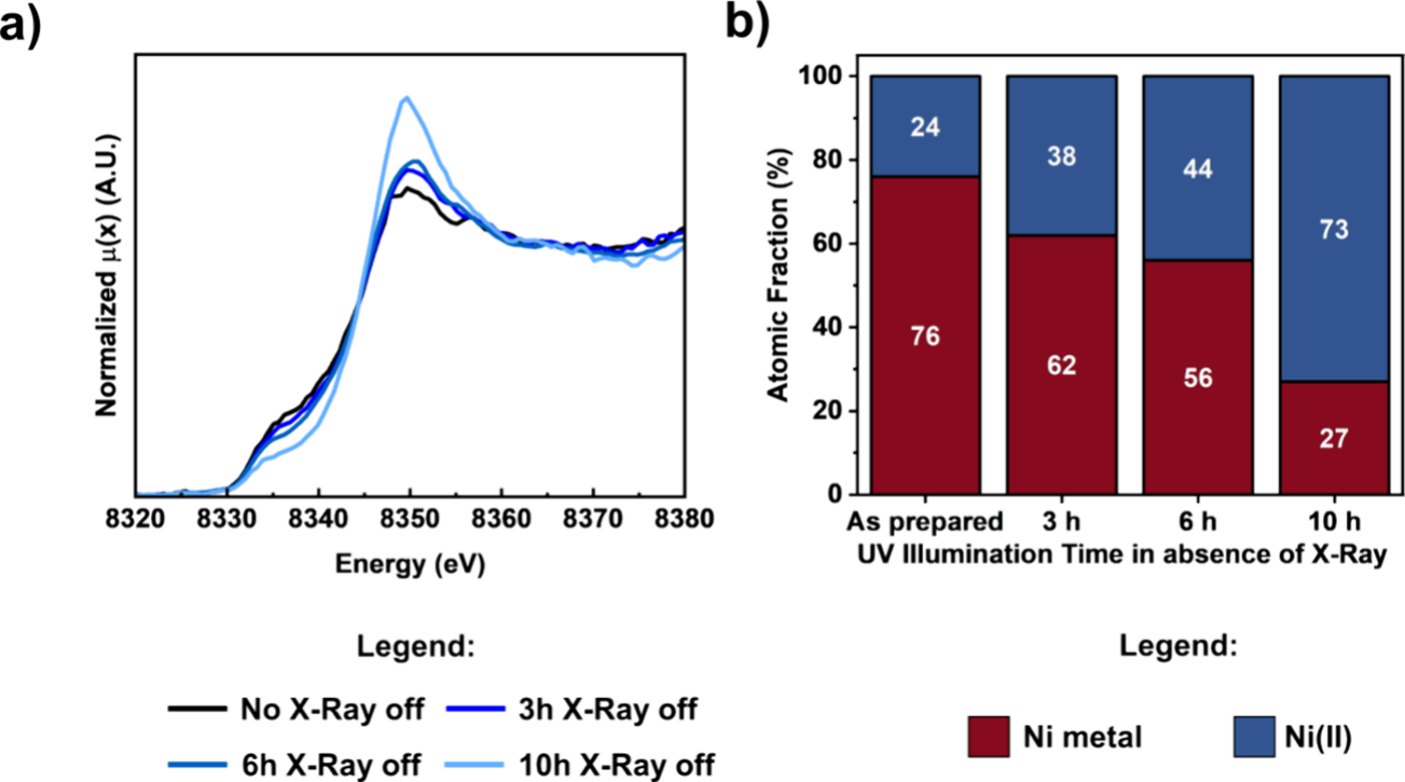
(a) Normalized Ni K-edge in situ spectra for different UV irradiation times in the absence of the X-ray probing beam. (b) Ni(0) and Ni(II) atomic fraction obtained by fitting the spectra in (a).

We show in Figure 7a in situ spectra measured at the end of the UV illumination period for different experiments carried out by systematically reducing the photocatalyst exposure to the probing beam under UV illumination (from continuous to no X-ray exposure). Figure 7b summarizes the results of the different in situ XAS experiments outlined in Figure S6 and Figure 6—indicating the Ni(0) and Ni(II) percentages after variable UV exposure time. Once more, it is evident that the shorter the exposure of the photocatalyst slurry to the probing X-ray beam, the more prominent the Ni oxidation. Taking the substoichiometric H_2_:O_2_ ratio into consideration, the Ni(0)/Ni(II) composition change can be explained by the oxidation of Ni(0) to Ni(II) phases by photogenerated holes (eq 5).
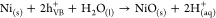
5

We propose that under UV illumination, metallic Ni sites trap and transfer photogenerated electrons to water, leading to H_2_ formation. Hence, in agreement with our earlier work,^15^ metallic Ni is the active phase responsible for hydrogen evolution—in contrast to that proposed in other previous studies.^16,27,31^ At the same time, other Ni sites (particles) are partially oxidized to NiO by photogenerated holes (i.e., h^+^ oxidizes Ni instead of H_2_O, eq 5), which results in a decrease of the hydrogen production rate over time (Figure 2b). The outer layer of the NiO phase produced under illumination is then hydrated, leading to the formation of a Ni(OH)_2_ phase (eq 6).
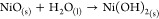
6

Finally, taking into account that Ni does not leach out from the photocatalyst (based on XPS and ICP-MS analyses), we considered the possibility that a dynamic equilibrium takes place under UV illumination where metallic Ni is oxidized by STO VB holes to soluble Ni(II) species. Such Ni(II)_(aq)_ species can then be either photoreduced by CB electrons to Ni, depositing onto the STO surface (i.e., photodeposition), or form Ni(OH)_2_ due to a local, relatively high pH.

Photodeposition of metal cocatalyst NPs on various photocatalysts under UV irradiation is well documented in the literature^40−42^ but typically requires a hole scavenger (e.g., methanol and ethanol).^43,44^

To test this possibility,^23^ we performed in situ XAS experiments with pristine STO powders suspended in DI water and in the presence of a dissolved Ni(II) salt (10 mM NiSO_4_ aqueous solution). Also in this case, we aimed at minimizing the exposure of the slurry to the probing beam, and hence, in situ XANES spectra were acquired only twice during the experiment, i.e., in the dark before starting UV illumination and directly after 4 h of UV irradiation. These spectra are shown in Figure S9, along with a reference spectrum of a NiSO_4_ aqueous solution taken in the absence of the STO photocatalyst. The in situ spectra for the STO slurry are identical before and after UV illumination and feature the same spectral shape of the reference NiSO_4_ aqueous solution. Thus, we can rule out the possibility of Ni^2+^ photoreduction to Ni(0) onto STO. Furthermore, GC analysis of the gas headspace (Figure S10) shows that the addition of the Ni(II) salt to the reaction medium does not lead to any increase of the H_2_ evolution rate but it rather has a detrimental effect on the photocatalytic H_2_ evolution rate of pristine STO. This result might be explained by the fact that solvated Ni(II) ions absorb UV photons, and hence attenuate the photon flux reaching STO.

**Figure 8:**
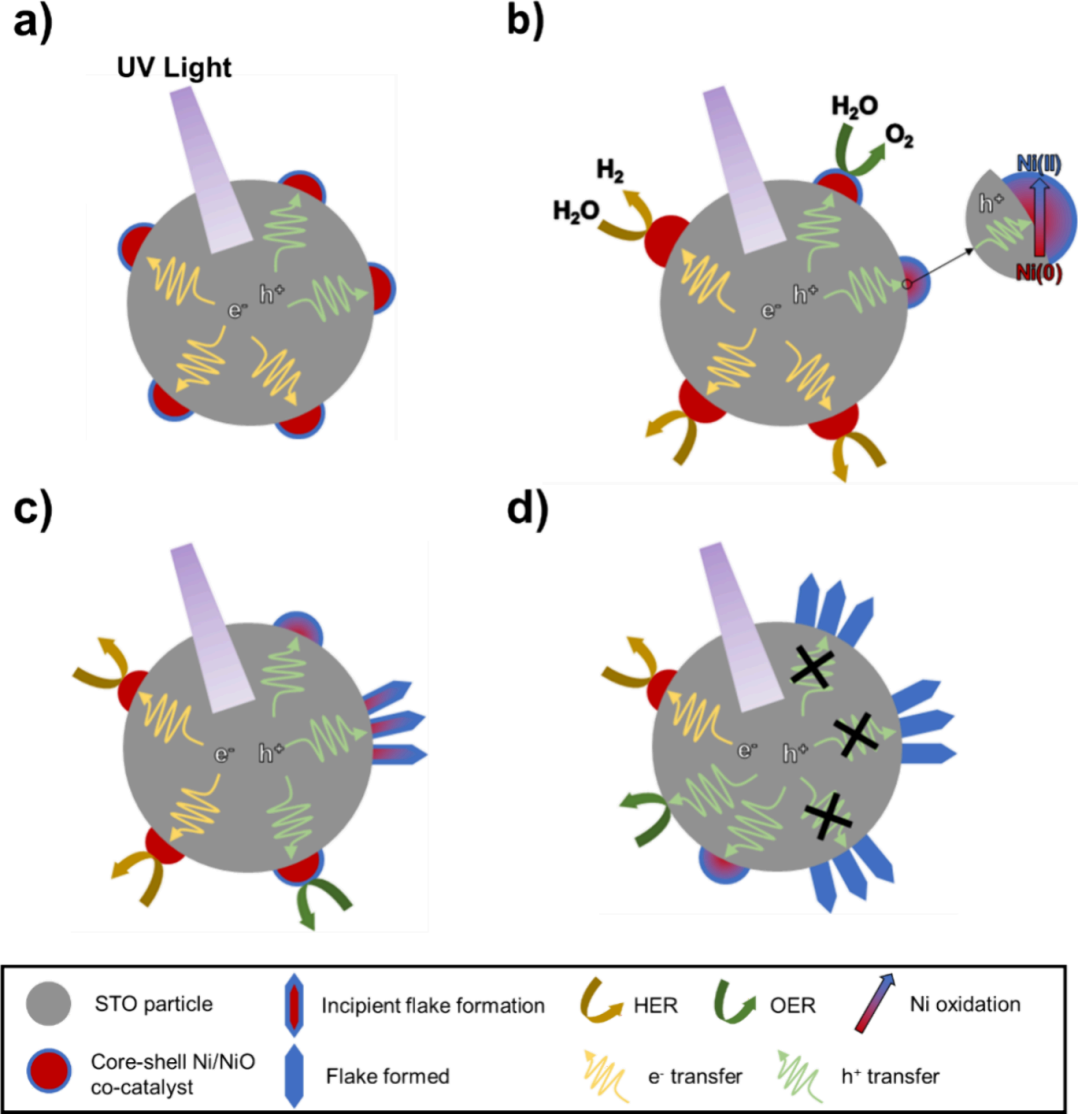
Schematic representation of the photocatalyst deactivation: (a) as-prepared photocatalyst and charge carrier generation upon UV excitation; (b) photocatalyzed HER, OER, and Ni oxidation to Ni(II); (c) restructuring of Ni cocatalyst; (d) partially deactivated photocatalyst after prolonged UV illumination.

To conclude, we sketch in Figure 8 the proposed mechanisms for photocatalyst water splitting and deactivation based on the results of in situ and ex-situ characterization techniques discussed above.

In the as-prepared state (Figure 8a), the Ni cocatalyst is in the form of Ni/Ni(II) core–shell NPs, with a Ni(0):Ni(II) at % ratio of approximately 4. Upon illumination, due to the interaction between (coupling of) the STO and the Ni NPs, charge carriers photogenerated in the STO can transfer to the Ni cocatalysts. We propose that in the early stage of UV illumination (Figure 8b), a portion of the Ni core–shell NPs can trap CB electrons and undergo reduction to the Ni(0) phase, according to the NiO (Ni^2+^)/Ni (Ni^0^) equilibrium potential at pH 7^45^ and the CB edge of STO (exit energy of CB electrons). Thus, the metallic Ni phase is the active site for water reduction, thus catalyzing HER. At the same time, VB holes can also transfer to cocatalyst NPs. Such holes can either be transferred to water, thus catalyzing the OER, or react with the Ni(0) phase, leading to Ni(0) oxidation. In the latter case, the oxidized Ni particles undergo morphological restructuring into a flake-like morphology (Figure 8c,d). By increasing the UV illumination time (Figure 8d), more core–shell particles are transformed into Ni oxide flake-like structures, which are inactive for water splitting. Different TEM and SEM images were taken for the same sample after photocatalysis for 6 h; see Figure S11. The different size of the flakes and morphology of the NPs provide evidence for the dynamic nature of the Ni-STO photocatalyst and can be ascribed to different stages of Ni oxidation. Eventually, this leads to water oxidation taking place at low rates (likely onto the bare STO surface), while some reduced cocatalyst particles keep acting as the H_2_ evolution site (Figure 8d).

Future studies should investigate the regeneration of the deactivated photocatalyst, e.g., by thermal treatment in reducing atmospheres, to reduce the high oxidation states of Ni to a metallic phase needed for hydrogen evolution. Even more desirable is to identify ways to avoid Ni oxidation. To achieve this, a possible strategy could be to deposit OER and HER cocatalysts on hole and electron-collecting facets, respectively, of the supporting semiconductor—a concept so far demonstrated only for noble metal-based cocatalysts—^46−48^ while for earth-abundant cocatalysts, stability issues remain a concern.

Finally, we investigated the effect of hole scavengers on the Ni-STO-photoinduced degradation. Adding hole scavengers, such as MeOH and EtOH, to the reaction medium is a common strategy to reduce the e^–^–h^+^ recombination rate, thus improving the photocatalyst performance in terms of hydrogen evolution rate.^49,50^ The data in Figure S12a show a significant improvement in the photocatalytic H_2_ evolution performance of the Ni-STO photocatalyst when MeOH is added to the solution as a hole scavenger. In a previous work, in situ XAS techniques were used to demonstrate the photoreduction of oxidized Ni phases (e.g., NiO) to metallic Ni (Ni^0^) on TiO_2_ photocatalysts in the presence of EtOH as a hole scavenger under UV illumination.^29^ The data in Figure S12a reveal a significantly higher and more stable hydrogen evolution rate (maximum value of 375 μmol h^–1^ g^–1^ compared to 50 μmol h^–1^ g^–1^) during a few hours of high photon flux UV illumination, confirming that (i) in the presence of a hole scavenger, the metallic Ni phase undergoes oxidation to a minor extent, hence sustaining a higher and almost constant H_2_ generation rate over time—in other words, inhibiting Ni oxidation increases the H_2_ generation rate; while (ii) in the absence of a hole scavenger, VB holes are responsible for Ni oxidation and for the degradation of the Ni-STO photocatalytic activity. Figure S12b,c shows an SEM image of the used Ni-STO photocatalyst tested in a methanol–water solution. The data indicate that the morphology of the Ni cocatalyst NPs remains unaltered compared to the as-prepared photocatalyst.

Interestingly, the schematic shown in Figure 8 is different from the structural changes proposed by Han and co-workers.^15^ Two differences should be noted in comparing the two studies, which are the following: (i) the sample was not exposed to a mild oxidation in air at 200 °C but carefully exposed to ambient conditions after thermal reduction, which might lead to a less conformal oxide shell coverage and a thinner oxide shell thickness, and (ii) the UV-light intensity used here was significantly higher than applied in the previous study.^15^ Future research should address the effect of the NiO/Ni(OH)_2_ shell thickness and illumination intensity on the performance and degradation of Ni-SrTiO_3_ photocatalysts in overall water splitting.

## Conclusions

In the present work, we used in situ XAS techniques to investigate the deactivation mechanism of a nanostructured Ni–STO photocatalyst for water splitting. We devised a liquid-phase spectroscopic cell for XAS measurements in fluorescence mode to monitor changes in the Ni cocatalyst oxidation state during photocatalytic H_2_ generation from photocatalyst suspensions in plain water. Continuous exposure of the photocatalyst to X-ray illumination was found to prevent the in situ oxidation of Ni during UV illumination. To overcome this effect (i.e., the undesired beam induced effect, likely due to photolysis of water), we carried out a series of in situ XAS experiments by systematically reducing the exposure of the UV-illuminated photocatalyst suspension to the probing beam. We demonstrated that the instability of the Ni cocatalyst is due to its oxidation and reconstruction, to Ni(II) species, caused by photogenerated VB holes. We concluded that this phenomenon is the root cause of the poor durability of Ni-STO water splitting photocatalysts. We also ruled out dissolution of Ni in the form of solvated Ni(II) species as well as photodeposition of solvated Ni(II) ions (via photoreduction) onto the photocatalyst surface.

## Supporting Information Available

Additional experimental details, and photocatalytic, XAS, ICP-MS, TEM, and SEM data (PDF)
