# Supplementary material for: In Situ X-ray Absorption Spectroscopy Study of the Deactivation Mechanism of a Ni-SrTiO3 Photocatalyst Slurry Active in Water Splitting
Source: J Phys Chem C Nanomater Interfaces. 2024 Sep 17;128(38):16020–31. doi: 10.1021/acs.jpcc.4c04688 (PMC11440603; doi:10.1021/acs.jpcc.4c04688)
Supplement: Supplementary file 1 — jp4c04688_si_001.pdf [file jp4c04688_si_001.pdf]

Supporting Information

In-situ X-ray Absorption Spectroscopy Study of the Deactivation Mechanism of a Ni-$SrTiO_3$ Photocatalyst Slurry Active in Water Splitting

MemetTursun Abudukade[a,†], Marco Pinna[a,b,c,†], Davide Spanu[b], Giuditta De Amicis[d], Alessandro Minguzzi[c,e], Alberto Vertova[c,e], Sandro Recchia[b], Paolo Ghigna[d], Guido Mul[a], Marco Altomare[a,*]

[a] *Department of Chemical Engineering, MESA+ Institute for Nanotechnology, University of Twente, P.O. Box 217, 7500 AE Enschede, The Netherlands*

[b] *Department of Science and High Technology, University of Insubria, Via Valleggio 11, 22100 Como, Italy*

[c] *Dipartimento di Chimica, Università degli Studi di Milano, Via Golgi 19, Milan 20133, Italy*

[d] *Dipartimento di Chimica, Università degli Studi di Pavia, Via Taramelli 16, Pavia 27100, Italy*

[e] *UdR INSTM di Milano - Consorzio Interuniversitario Nazionale per la Scienza e Tecnologia dei Materiali – INSTM, Via G. Giusti 9, 50121, Firenze, Italy*

* Corresponding author. Email: m.altomare@utwente.nl

† Authors have equal contributions

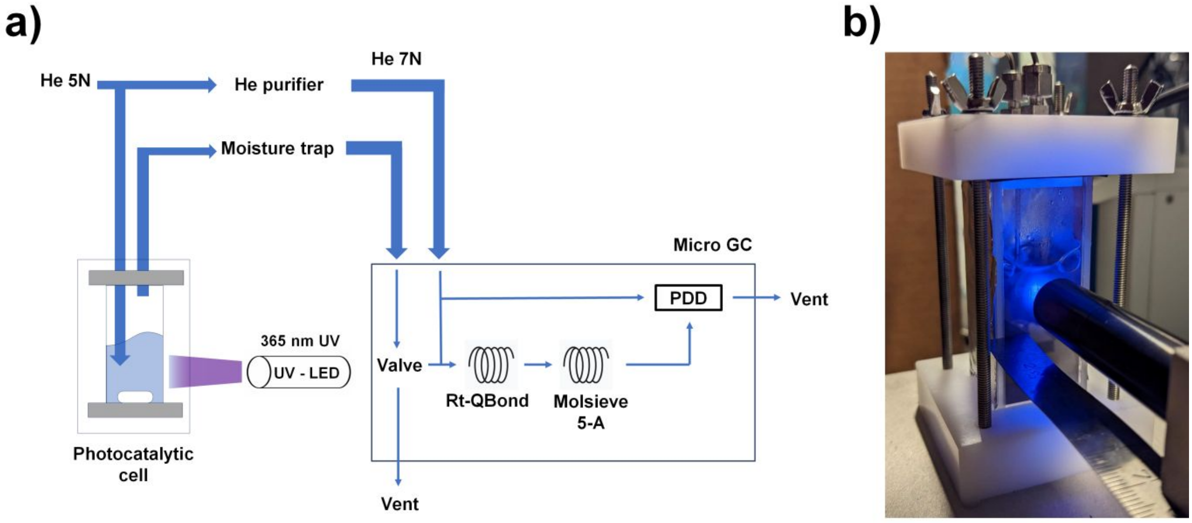

Figure S1. (a) Schematic of the photocatalytic setup and (b) photography of the photocatalytic cell under UV illumination.

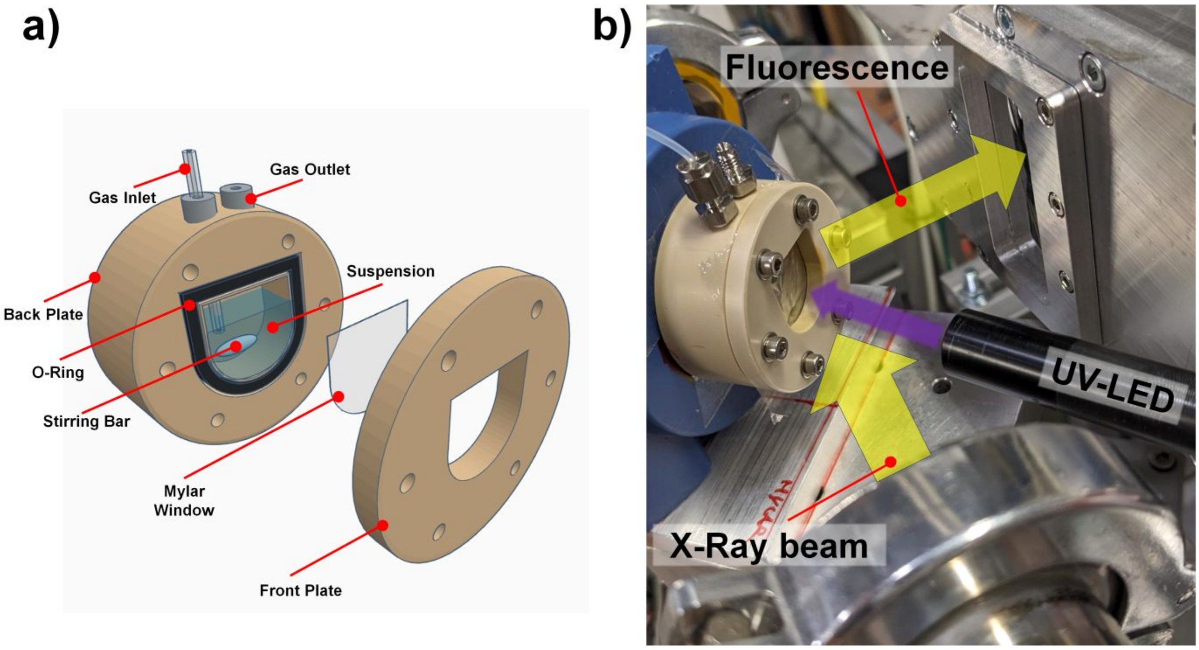

Figure S2. (a) Schematic of in-situ XAS cell, and (b) photograph of the in-situ XAS setup used at ELETTRA.

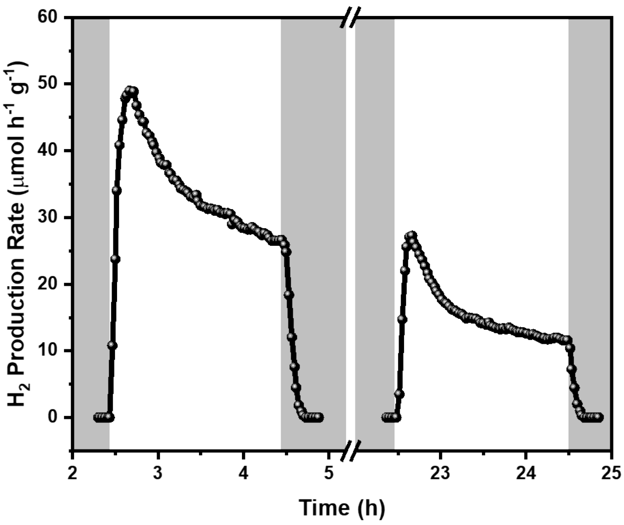

Figure S3: Production rate profiles for H_2 during two consecutive photocatalytic irradiation steps. The grey areas represent times in the absence of UV illumination (dark condition) while the white ones represent UV irradiation steps.

Table S1. Surface atomic content of Ni (at%) as determined from XPS measurements for the samples before and after photocatalysis.

	Ni surface atomic content (at%)
Before photocatalysis	16.8
After photocatalysis	16.3

Table S2. ICP-MS measurements of filtered suspension sampled at different times during the photocatalytic tests. Results are reported as percentage of dissolved Ni relative to the nominal amount of Ni co-catalyst initially loaded on STO.

Initial purging time (hours)	UV illumination time (hours)	Final purging time (hours)	Dissolved Ni content (wt. %)*
15 h	-	-	0.022
15 h	6.5 h	-	0.017
15 h	6.5 h	1 h	0.038
2.5 h	-	-	0.108
2.5 h	6.5 h	-	0.016
2.5 h	6.5 h	1 h	0.024

*The dissolved Ni content was calculated according to Equation S1:

$$\text{Dissolved Ni content (wt.\%)} = \frac{\text{Dissolved Ni mass in water}}{\text{Initial Ni mass on STO}} * 100 \qquad \text{(S1)}$$

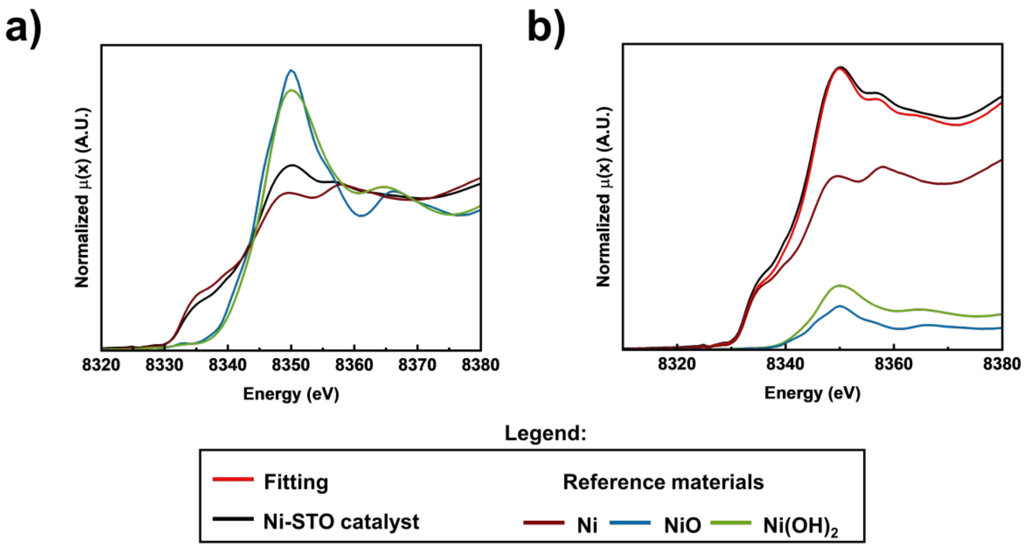

Figure S4. (a) XANES spectra of as-prepared Ni-STO, and of metallic Ni, NiO, and $Ni(OH)_2$ standards (used as fitting components); (b) linear combination fitting of the spectrum of the as-prepared Ni-STO photocatalyst.

The spectrum of metallic Ni is characterized by a large shoulder between 8330 and 8335 eV, arising from transition from the 1s orbital to empty p states of the conduction band. In this energy range, the spectra of Ni(II) compounds show very small intensity, which is due to dipole

forbidden 1s to 3d transitions. The largest intensity in the spectra of Ni(II) compounds is found at ca. 8350 eV, where dipole allowed transitions are found, while at this energy the spectrum of metallic Ni shows smaller oscillations which are structural in origin.

For these and all other XAS spectra, as constraints for fitting we fixed the weights of different Ni phases to have a positive value, and their sum to be equal to 1.

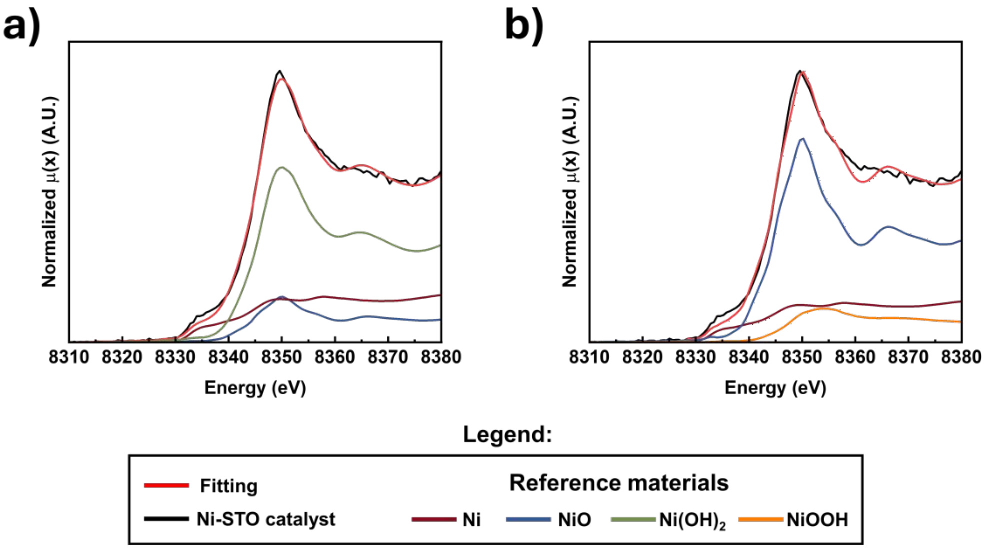

Figure S5: Different linear combination fittings of a spectrum obtain under illumination of the Ni-STO photocatalyst slurry, Ni, NiO, and $Ni(OH)_2$ (a) or Ni, NiO, and NiOOH (b) respectively, were used as fitting components.

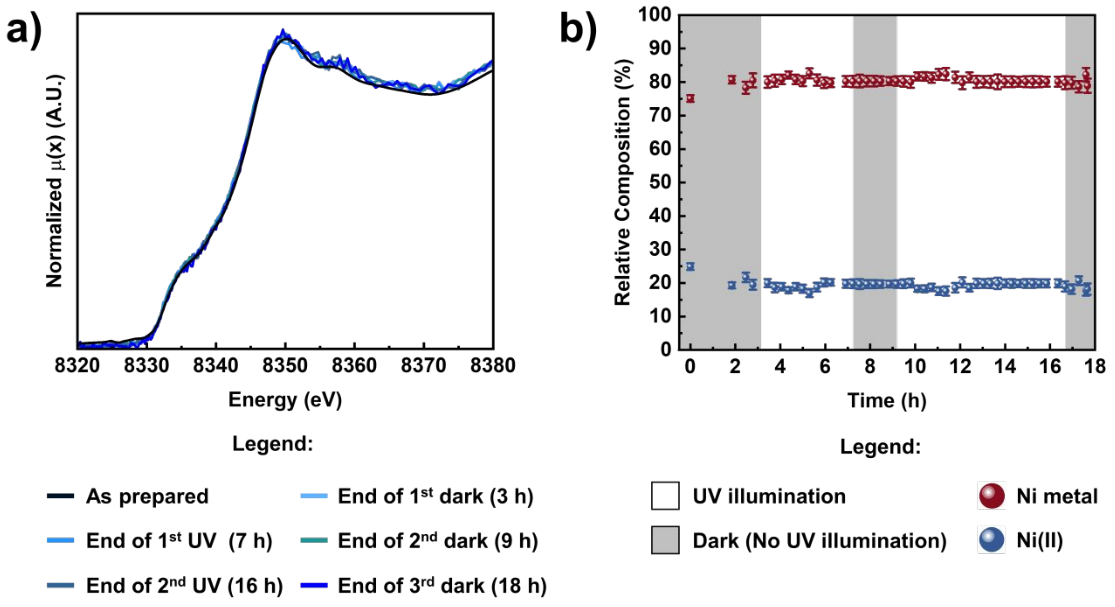

Figure S6. (a) Normalized Ni K-edge XANES spectra of Ni-STO under intermittent UV illumination. "Dark" and "UV" refer respectively to dark or UV illumination conditions. (b) Fitting results of in-situ XANES spectra of Ni-STO. The data at t = 0 refer to ex-situ XANES measurements of the as-prepared Ni-STO photocatalyst, grey areas represent data points acquired under dark conditions, while white areas refer to data points acquired under UV illumination.

The absence of changes in Ni phase composition under continuous X-ray exposure (Figure S6) can be explained by X-ray beam damage. Various studies in the literature show that beam damage has often a reducing effect.[1–3] In aqueous media, high brilliance X-rays are reported to cause water radiolysis according to Equation S2 [4]:

$$H_2O_{(l)} \xrightarrow{h\nu} e^-_{(aq)} + H_2O^{\cdot+}_{(aq)} \qquad (S2)$$

The solvated free electrons can react with the photogenerated valence band holes, hence preventing photo-oxidation of the Ni co-catalyst to Ni(II) phases [3].

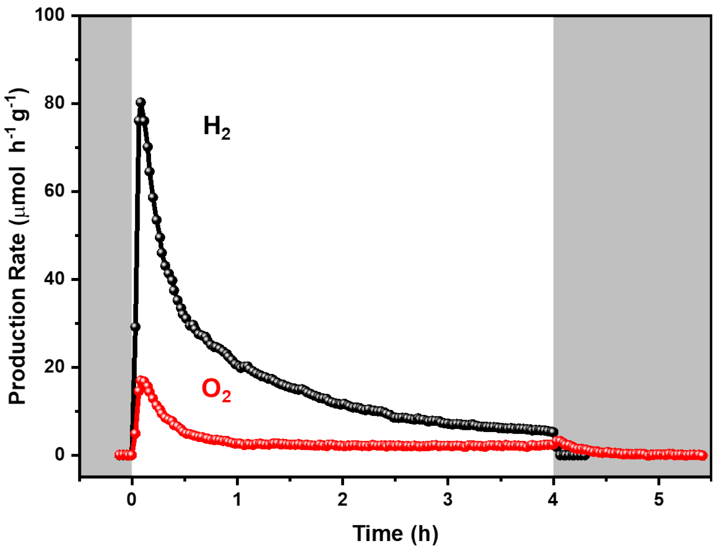

Figure S7: Production rate profiles for H_2 (black) and O_2 (red) during overall photocatalytic water splitting carried out in the in-situ XAS cell in the absence of X-rays. The grey areas represent times in the absence of UV illumination (dark condition) while the white ones represent UV irradiation steps.

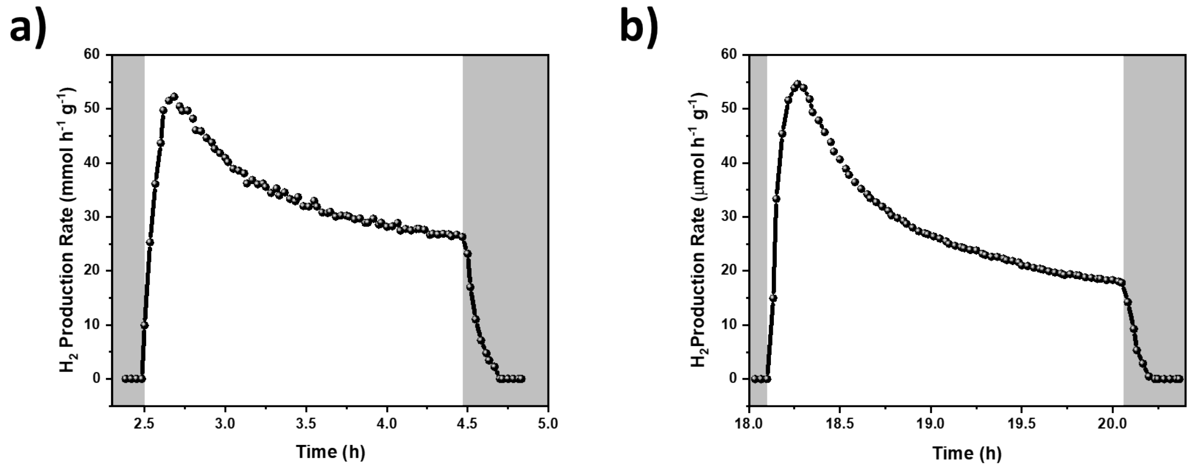

Figure S8: H_2 production rate profiles for two photocatalytic experiments carried out after different durations of the initial He purging step: (a) dark for 2.5 h; (b) dark for 18 h. t = 0 h is when the catalyst was suspended in DI water. Grey areas represent data points acquired under dark conditions, while white areas refer to data points acquired under UV illumination.

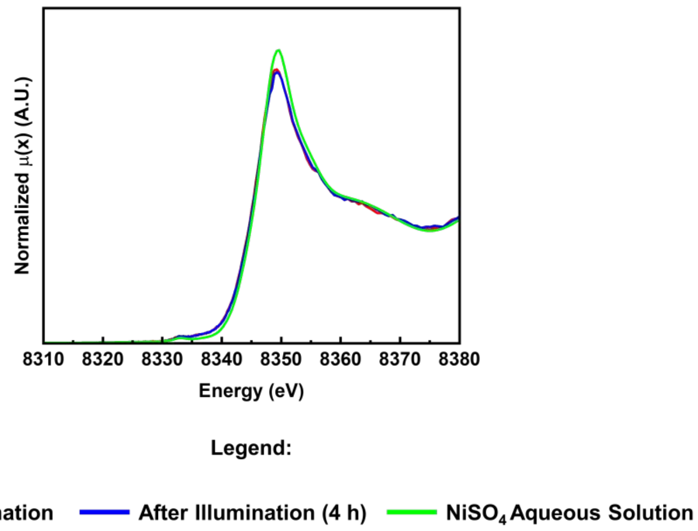

Figure S9. XANES spectrum at the Ni K-edge of $NiSO_4$ (aq.) reference and in-situ spectra for pristine STO in 10 mM $NiSO_4$ solution under UV illumination.

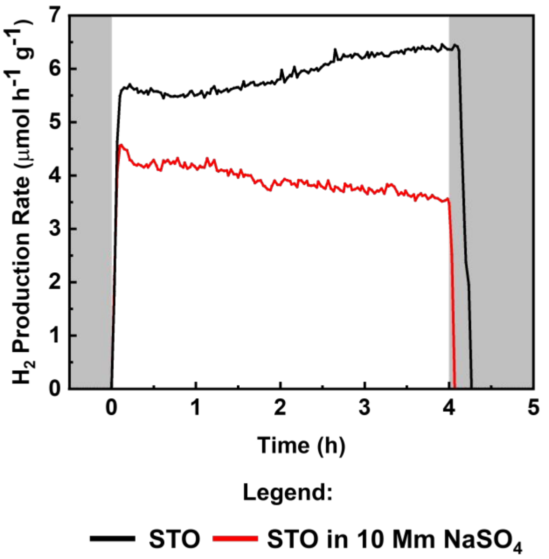

Figure S10. H_2 evolution during photocatalytic water splitting with pristine STO, with and without $NiSO_4$ salt. Grey areas represent data points acquired under dark conditions, while white areas refer to data points acquired under UV illumination.

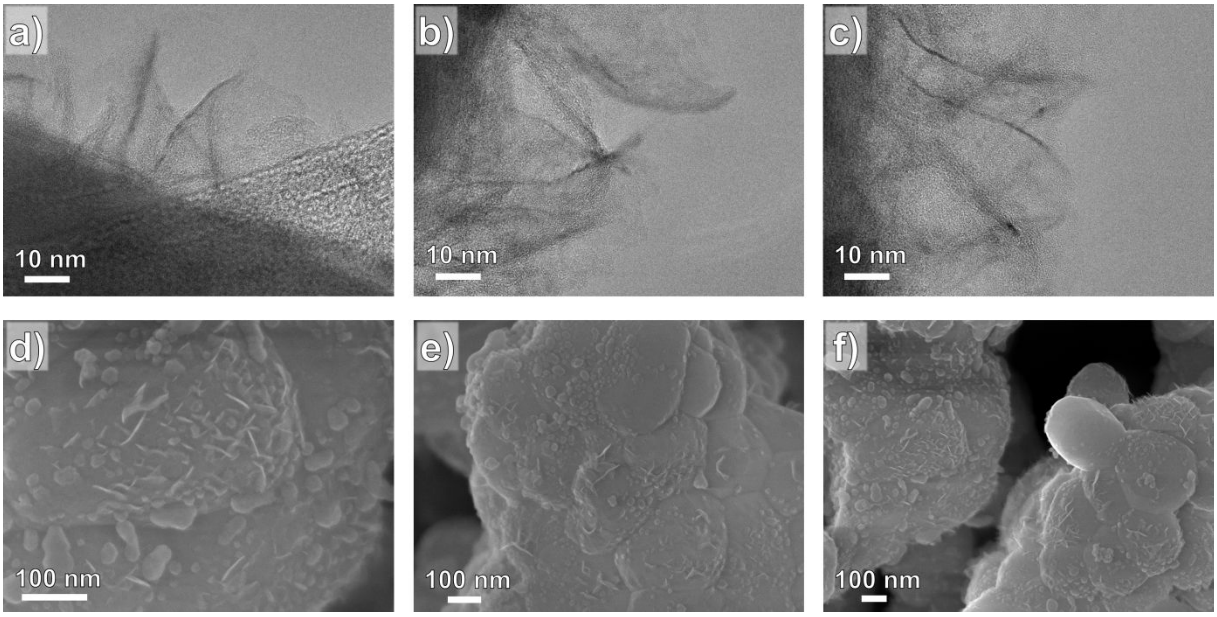

Figure S11. (a-c) TEM and (d-f) SEM images of Ni-STO photocatalyst after 6 hours of photocatalysis.

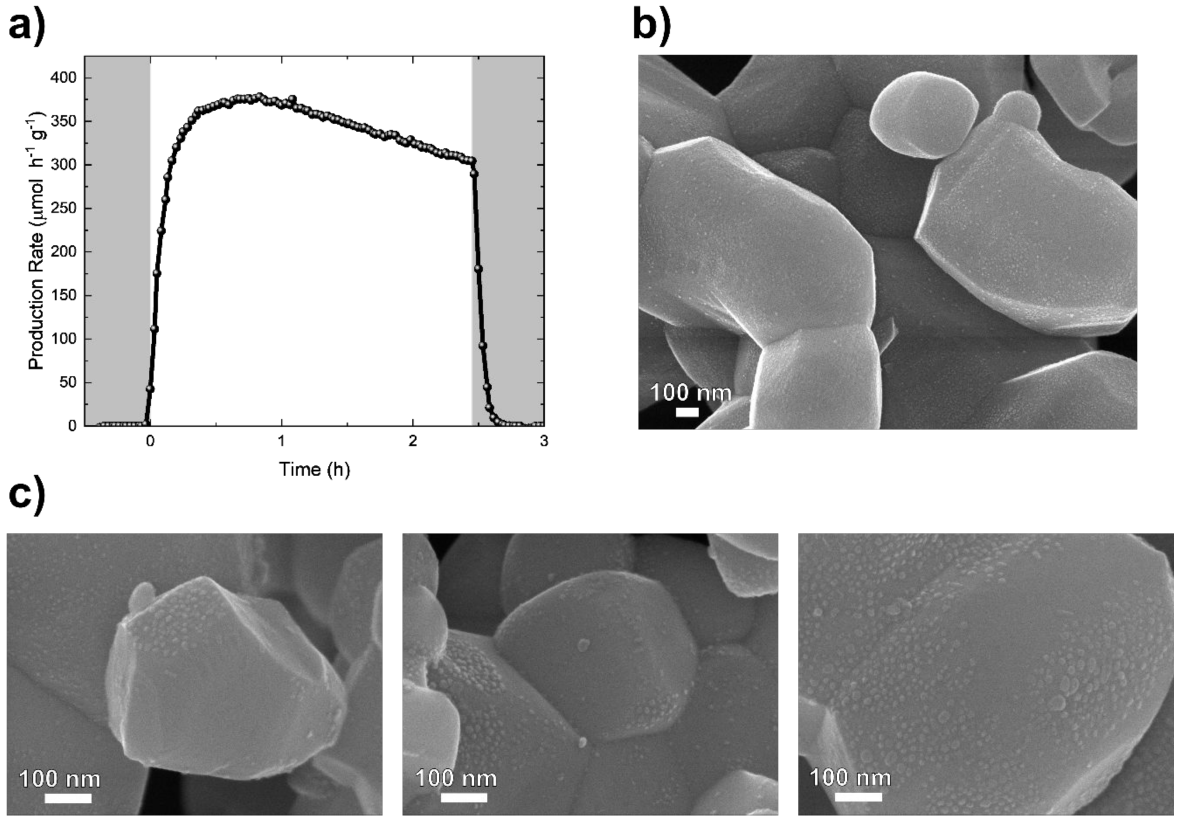

Figure S12. (a) H_2 production rate over time for Ni-STO under photocatalytic alcohol reforming conditions (in a 20 vol% MeOH/H_2O mixture), (b,c) High-resolution SEM (HR-SEM) images of Ni-STO after 6 h of photocatalysis in MeOH/H_2O mixture.

References

(1) Mesu, J. G.; Beale, A. M.; De Groot, F. M. F.; Weckhuysen, B. M. Probing the Influence of X-Rays on Aqueous Copper Solutions Using Time-Resolved in Situ Combined Video/X-Ray Absorption near-Edge/Ultraviolet-Visible Spectroscopy. *Journal of Physical Chemistry B* **2006**, *110* (35), 17671–17677. https://doi.org/10.1021/JP062618M/SUPPL_FILE/JP062618MSI20060712_023218.PDF.

(2) Laverne, J. A.; Pimblott, S. M. New Mechanism for H2 Formation in Water. *Journal of Physical Chemistry A* **2000**, *104* (44), 9820–9822. https://doi.org/10.1021/JP002893N/ASSET/IMAGES/LARGE/JP002893NF1.JPEG.

(3) Zabilska, A.; Clark, A. H.; Ferri, D.; Nachtegaal, M.; Kröcher, O.; Safonova, O. V. Beware of Beam Damage under Reaction Conditions: X-Ray Induced Photochemical Reduction of Supported VO_x Catalysts during *in Situ* XAS Experiments. *Physical Chemistry Chemical Physics* **2022**, *24* (36), 21916–21926. https://doi.org/10.1039/D2CP02721F.

(4) George, G. N.; Pickering, I. J.; Pushie, M. J.; Nienaber, K.; Hackett, M. J.; Ascone, I.; Hedman, B.; Hodgson, K. O.; Aitken, J. B.; Levina, A.; Glover, C.; Lay, P. A. X-Ray-Induced Photo-Chemistry and X-Ray Absorption Spectroscopy of Biological Samples. *J Synchrotron Radiat* **2012**, *19* (6), 875–886. https://doi.org/10.1107/S090904951203943X.
